# Host and tumor derived MMP13 regulate extravasation and establishment of colorectal metastases in the liver

**DOI:** 10.1186/s12943-014-0282-0

**Published:** 2015-02-22

**Authors:** Alisha M Mendonsa, Michael N VanSaun, Alessandro Ustione, David W Piston, Barbara M Fingleton, David Lee Gorden

**Affiliations:** Department of Cancer Biology, Vanderbilt University, 2220 Pierce Ave S, Nashville, TN 37232 USA; Department of Surgery, Vanderbilt University, 801 Oxford House, 1313 21st Ave. S, Nashville, TN 37212 USA; Department of Molecular Physiology and Biophysics, Vanderbilt University, 702 Light Hall 21st Avenue South, Nashville, TN 37232 USA

**Keywords:** NAFLD, Liver, Steatosis, Metastasis

## Abstract

**Background:**

Non alcoholic fatty liver disease (NAFLD) is one of the most common liver diseases in the United States and worldwide. Our studies have previously shown an increase in metastatic burden in steatotic vs. normal livers using a mouse model of diet induced steatosis. In the present study we aim to identify and evaluate the molecular factors responsible for this increase in tumor burden.

**Methods:**

We assessed changes in expression of a panel of matrix metalloproteinases (MMPs) using qRT-PCR between normal and steatotic livers and validated them with western blot analysis of protein levels. To evaluate the role of MMP13 on tumor development, we utilized a splenic injection model of liver metastasis in Wildtype and *Mmp13* deficient mice, using either parental or stable *Mmp13* knockdown cell lines. Further, to evaluate changes in the ability of tumor cells to extravasate we utilized whole organ confocal microscopy to identify individual tumor cells relative to the vasculature. MTT, migration and invasion assays were performed to evaluate the role of tumor derived MMP13 on hallmarks of cancer *in vitro.*

**Results:**

We found that MMP13 was significantly upregulated in the steatotic liver both in mice as well as human patients with NAFLD. We showed a decrease in metastatic tumor burden in *Mmp13*−/− mice compared to wildtype mice, explained in part by a reduction in the number of tumor cells extravasating from the hepatic vasculature in the *Mmp13*−/− mice compared to wildtype mice. Additionally, loss of tumor derived MMP13 through stable knockdown in tumor cell lines lead to decreased migratory and invasive properties *in vitro* and metastatic burden *in vivo*.

**Conclusions:**

This study demonstrates that stromal as well as tumor derived MMP13 contribute to tumor cell extravasation and establishment of metastases in the liver microenvironment.

**Electronic supplementary material:**

The online version of this article (doi:10.1186/s12943-014-0282-0) contains supplementary material, which is available to authorized users.

## Introduction

The obesity epidemic has been closely linked with an increased incidence of non alcoholic fatty liver disease (NAFLD) [1]. NAFLD results from accumulation of fat in the liver which alters the local liver microenvironment leading to changes in lipid profiles, recruitment of inflammatory cells and changes in cytokine expression [2,3]. The changes in the tissue microenvironment can affect cell-cell interactions and influence the development of both primary and metastatic tumors. Epidemiological studies show that NAFLD has been linked to an increase in the risk for development of primary liver cancer [4,5] but very little is known about the effect of steatosis on tumor metastasis to the liver. The liver is a frequent site of metastasis for several cancers such as colorectal cancer, breast and pancreatic cancer. In addition, obesity is an independent risk factor for the development of these tumor types among others [6]. Previous studies from our lab have shown that mice with high fat diet induced steatosis have an increase in metastatic burden compared to mice with normal livers [7]. Metastasis represents the end-stage of cancer progression and is responsible for most cancer related deaths [8]. Improved understanding of the metastatic tumor microenvironment is important in devising therapies to impact this stage of the disease.

Matrix metalloproteinases (MMPs) are a family of zinc dependent proteases that are capable of cleaving various components of the extracellular matrix. Apart from matrix molecules, they can also cleave adhesion proteins, activate growth factors such as TGF-β and VEGF as well as release and activate cytokines [9,10]. MMPs are produced by tumor cells, stromal cells and infiltrating inflammatory cells and facilitate host-tumor interactions. Members of the MMP family have been associated with progression through multiple stages of cancer, from initiation to acquisition of metastatic properties [11,12]. However, MMPs have also been shown to have anti-tumorigenic functions and are important for normal developmental and wound healing responses [13]. Early clinical trials broadly targeting the MMP family as a whole, led to unintended clinical side effects. Improved understanding of specific roles for individual MMPs and development of pharmaceutical agents that selectively target individual MMPs may be part of effective therapeutic strategies in the future [14].

MMP13 is an interstitial collagenase that is capable of cleaving multiple collagens, preferentially collagen II, as well as other matrix substrates such as gelatin, fibronectin, and aggrecan relevant to tumor metastasis [15]. This protease has been previously linked to the progression of fibrotic liver disease [16]. In addition, increased expression of MMP13 has been associated with poor prognosis in patients with colorectal cancer metastasis to the liver [17]. MMP13 was identified as a part of the breast cancer metastasis signature and was associated with decreased overall survival and metastasis in breast cancer and renal cell carcinoma [18-20]. Furthermore, stroma-derived MMP13 was found to be involved in the growth of liver, lung, brain and heart metastases of melanoma cells [21]. We thus hypothesize that both stromal and tumor derived MMP13 play an important role in modulating the liver microenvironment and facilitate the establishment of liver metastasis.

## Results

### MMP13 expression is elevated in the steatotic liver

**Figure 1:**
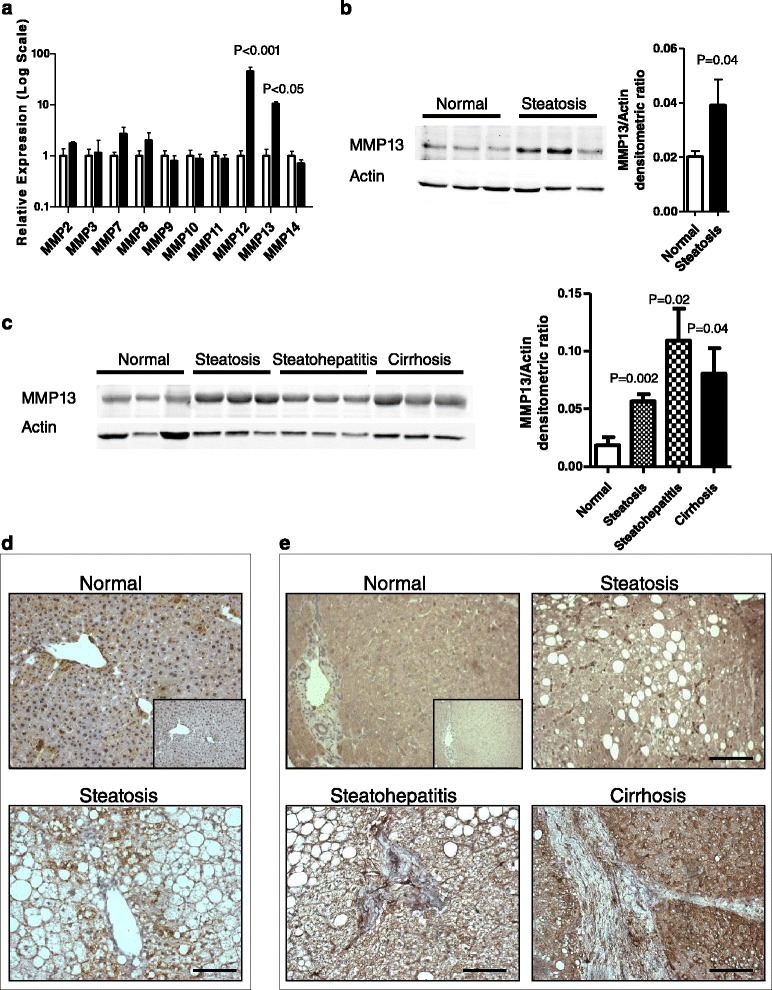
**MMP13 is elevated in the steatotic liver. (a)** Relative gene expression of matrix metalloproteinases (MMPs) in liver tissue of mice with steatosis (black bars) compared to normal (white bars) mice. Transcript levels were normalized to GAPDH and expressed as fold change relative to normal controls. Values represent the mean (n = 3 per group). Statistical analysis was carried out using 2-way ANOVA followed by Bonferroni post test between normal and steatotic samples for each MMP tested. **(b)** Representative western blot showing 3 samples per group and densitometric quantification (n = 9 per group) of MMP13 protein levels relative to actin levels in livers of wildtype C57bl/6 mice with and without steatosis. Statistical analysis was carried out using a 2-tailed t-test, P = 0.04. **(c)** Representative western blot and densitometric quantification of MMP13 protein levels relative to actin levels in human liver samples through multiple stages of NAFLD (n = 9 per group). Statistical analysis was carried out using a 2-tailed t-test with respect to normal livers. No significant differences were observed between the different stages of progression of NAFLD. **(d)** Representative immunohistochemical (IHC) staining of MMP13 in murine normal and steatotic livers. Inset represents IgG control. **(e)** Representative IHC of human MMP13 at different stages of NAFLD. MMP13 staining is detected at low levels in the normal liver hepatocytes and is increased in the stromal cells of livers with steatosis and steatohepatitis. Additionally, MMP13 is present in select hepatocytes in cirrhotic livers. Images were taken at 20X, Scalebar represents 100 microns.

Previous studies in our lab have found a significant increase in the metastatic tumor burden to the liver in the setting of steatosis as compared to normal mouse livers [7]. Additionally, our group has identified MMP9 as an important mediator of tumor metastasis to the liver [22] and other groups have shown several additional MMPs to be important in metastasis [23]. With the increasing recognition of NAFLD as a significant liver disease, it is highly relevant to understand which MMPs are altered in the steatotic microenvironment and whether these MMPs contribute to the increased metastasis. We assessed the relative gene expression levels of a panel of MMPs, associated with tumor progression, in the liver tissue of mice with steatosis compared to normal mice (n = 3). We found that *Mmp12* (P < 0.001) and *Mmp13* (P < 0.05) were significantly upregulated in the liver of mice with steatosis compared to normal livers using 2-way ANOVA followed by the Bonferroni post-test (Figure 1a). Both *Mmp12* and *Mmp13* have been recently shown to be elevated in the liver in other models of diet induced obesity as well [24]. MMP13 has previously been associated with fibrotic liver disease [16] and its expression in tumors has been associated with poor prognosis in patients with colorectal cancer metastasis to the liver [17]. MMP13 protein levels were evaluated in the mouse liver and were found to be increased in the steatotic livers compared to normal livers (n = 9, P = 0.04, using the 2-tailed t-test)(Figure 1b). To determine whether changes in MMP13 levels were relevant to the human progression of NAFLD, we evaluated the MMP13 protein levels by western analysis from liver lysates of normal livers and compared them to livers with steatosis, steatohepatitis or NAFLD related cirrhosis. Figure 1c shows that MMP13 protein expression is increased with NAFLD (P < 0.05, n = 9 per group, using 2-tailed t-test compared to normal livers). No significant differences were observed between the different stages of NAFLD. Immunohistochemical analysis of MMP13 expression shows staining of MMP13 with steatosis in the murine liver (Figure 1d) and varied distribution of the protein in both stromal cells as well as hepatocytes in human patient samples with NAFLD (Figure 1e). Thus, MMP13 is elevated both in human NAFLD as well as in a mouse model of diet induced steatosis.

### Loss of host derived MMP13 leads to decreased tumor metastasis to the liver

**Figure 2:**
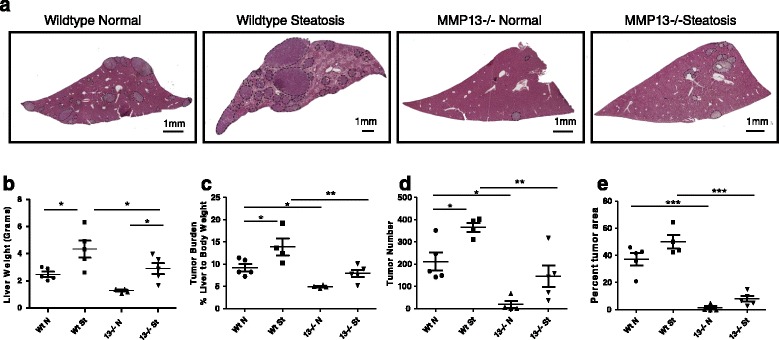
**Loss of host derived MMP13 leads to decreased tumor metastasis to the liver. (a)** Representative liver cross sections stained with Haematoxylin & Eosin of wildtype and MMP13−/− mice with normal or steatotic livers show an increased tumor burden in wildtype mice compared to MMP13−/− mice (n = 5). Tumors are denoted by black dashed line. Quantification of metastatic tumor burden by **(b)** liver weight, **(c)** tumor burden measured as percent liver weight to total body weight, metastatic seeding by quantification of **(d)** tumor number and **(e)** tumor area per liver section in normal and steatotic livers. Statistical analysis was performed using GraphPad Prism software. Data was analyzed by using one-way ANOVA followed by Newman-Keuls Multiple Comparison Test. P values are represented by stars where: * ≤ .05, ** ≤ .01, and *** ≤ .001.

Elevation of MMP13 in the steatotic liver suggested that it could play an important role in priming the steatotic liver microenvironment for tumor establishment. To elucidate the role of host MMP13 on tumor metastasis to the liver, *Mmp13* null (*Mmp13*−/−) mice were fed a high fat diet to induce steatosis. *Mmp13*−/− mice developed steatosis comparable to the wildtype counterparts (Additional file 1: Figure S1). Subsequently, wildtype and *Mmp13*−/− mice with and without steatosis were inoculated with 5 × 10^5^ syngeneic MC38 colon cancer cells through the intrasplenic/portal route to generate experimental liver metastases. After 2 weeks, mice were sacrificed and livers were harvested. The liver weight and percent liver weight to total body weight ratios were measured to determine metastatic burden (Figure 2b, c), and livers were fixed, dissected and stained by H&E (Figure 2a) to record the incidence (Figure 2d) and area (Figure 2e) of metastases. Tumor burden and incidence of metastases increased in the steatotic compared with normal mice in both the wildtype and *Mmp13*−/− mice. However, the tumor burden, incidence and area of liver metastases was markedly reduced in the *Mmp13*−/− mice compared to the wildtype mice after tumor cell injection, thus supporting the role of MMP13 in metastatic tumor growth to the liver both in normal and steatotic mice. Statistical analysis was carried out by one-way ANOVA followed by Newman-Keuls multiple comparison test. P values are represented by stars where: * ≤ .05, ** ≤ .01, and *** ≤ .001 (Figure 2).

Additionally, liver metastases were stained for Ki67 (proliferation marker) and cleaved caspase-3 (apoptosis marker) to further examine the role of MMP13 in promoting the proliferation and outgrowth of metastatic tumors (Additional file 1: Figure S2). Using one-way ANOVA analysis, no significant differences were observed in the percentage of tumor cells proliferating or undergoing apoptosis between the wildtype and *Mmp13*−/− tumors of comparable size suggesting no difference in the rate of tumor growth between the WT and *Mmp13*−/− mice. Additionally, there was no significant difference in tumor vascularity as determined by vWF staining (Additional file 1: Figure S2). To determine whether there were changes in tumor outgrowth, a histogram of tumor size distribution were plotted (Additional file 1: Figure S3). *Mmp13*−/− mice had fewer tumors and the distribution curve shifted to the left compared to WT mice, however the tumors were still capable of becoming large, suggesting that loss of host MMP13 could affect some of the earlier steps in the metastatic cascade such as tumor cell survival in the vasculature and seeding to the liver or ability of tumor cells to adhere to the vasculature and extravasate into the liver tissue.

### Loss of host MMP13 affects the ability of tumor cells to extravasate

**Figure 3:**
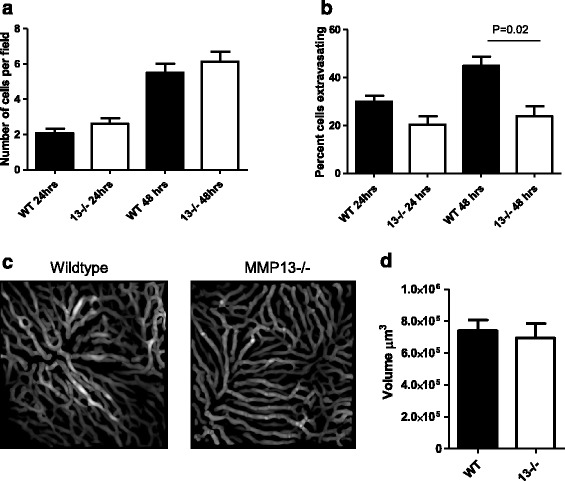
**Lack of stromal MMP13 leads to decreased tumor cell extravasation from the microvasculature. (a)** Number of tumor cells in the liver at 24 and 48 hours post intrasplenic injection of MC38 tumor cells quantified as average number of cell tracker red labeled cells per 10X field from 5 random images per mouse (n = 3). **(b)** Quantification of the percentage of tumor cells extravasating in the liver at 24 and 48 hours post injection of wildtype and MMP13−/− mice (n = 3). Statistical analysis was performed using the 2-tailed t-test. **(c)** Representative image of 3D reconstruction of wildtype and MMP13−/− hepatic vasculature and **(d)** quantification of hepatic vascular volume in wildtype and MMP13−/− mice (n = 5). Statistical analysis was performed using the 2-tailed t-test.

Since we observed a decrease in tumor burden with the loss of MMP13 in both normal and steatotic mice, we focused on the role of MMP13 on tumor metastasis to the liver irrespective of the diet. To determine whether the decrease in tumor burden in the *Mmp13*−/− mice compared to wildtype mice resulted from a difference in the ability of the tumor cells to survive in circulation and seed the liver, we injected normal wildtype or *Mmp13*−/− mice with 1 × 10^6^ MC38 tumor cells, labeled with cell tracker red, and sacrificed the mice 24 and 48 hours post injection. Livers were harvested, processed, sectioned and the number of tumor cells per section were quantified (Figure 3a). These results demonstrate no significant differences in the number of tumor cells present in the livers of these mice of wildtype and *Mmp13*−/− mice at 24 (P = 0.2) and 48 (P = 0.4) hours post injection and therefore no difference in survival or seeding of the cells to the liver using the two-tailed student t test.

To investigate the role of host MMP13 on the ability of MC38 colon cancer cells to extravasate from the vasculature, we adapted the methodology developed by Martin et al. [25]. MC38 cells were labeled with cell tracker red and injected into normal wildtype or *Mmp13*−/− mice. Mice were sacrificed at 24 and 48 hours post injection, their livers perfused with saline and their vasculature labeled with tomato lectin. The liver explants were then imaged using a confocal microscope to visualize individual tumor cells relative to the vasculature and compare the percentage of tumor cells that had extravasated in wildtype versus *Mmp13*−/− mice at each time point. We observed a decrease in the percentage of tumor cells that had extravasated in the MMP13−/− mice compared to that of the wildtype mice which was not significant at 24 hours (P = 0.06 using 2-tailed t-test) but became significant at 48 hours(P = 0.02 using 2-tailed t-test) post tumor cell injection (Figure 3b).

The hepatic vascular volume was evaluated from the 3-D vascular reconstructions (Figure 3c, d) of wildtype and *Mmp13*−/− mice liver. No difference in quantity of vascular staining was observed between the wildtype and *Mmp13*−/− mice indicating that changes in extravasation do not result from changes to the vascular capacity (P = 0.7 using 2-tailed t-test).

### Loss of tumor derived MMP13 leads to decrease in migratory and invasive properties of cells in vitro

**Figure 4:**
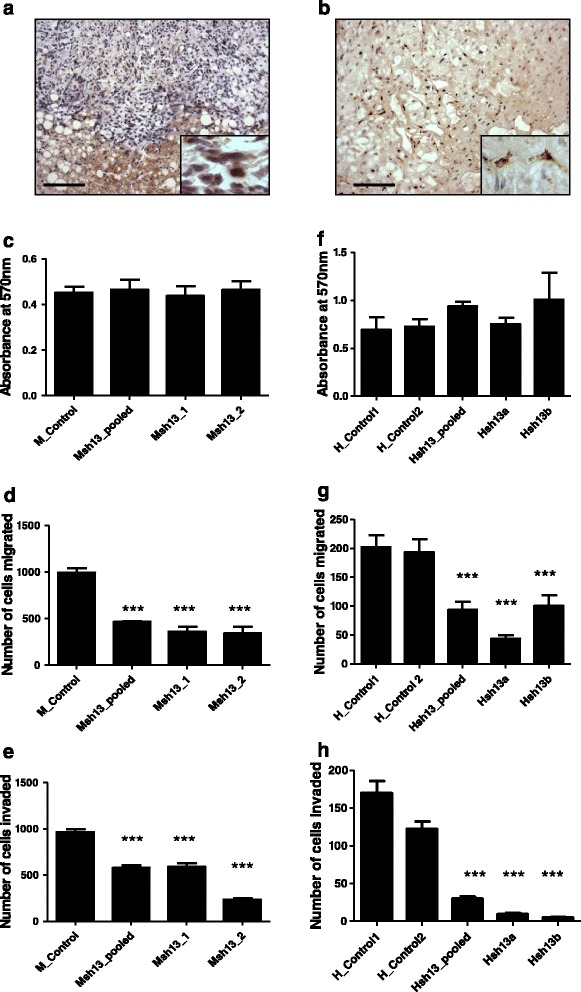
**Effect of knockdown of MMP13 in tumor cells on various hallmarks of cancer** ***in vitro*** **.** Immunohistochemical staining for MMP13 in representative sections of **(a)** murine MC38 tumor in the mouse liver and **(b)** human colorectal cancer metastasis to the liver at 20X magnification. Scalebar represents 100 microns. Inset shows individual tumor cells at 63X magnification. MTT assay shows no change in proliferation after loss of MMP13 *in vitro* in **(c)** MC38 or **(f)** HCT116; control and MMP13 knockdown cell lines. Knockdown of MMP13 leads to decreased ability of tumor cells to migrate *in vitro* as determined by a transwell migration assay in **(d)** MC38 and **(g)** HCT116 cell lines. Knockdown of MMP13 leads to decreased ability of tumor cells to invade *in vitro* as determined by the transwell invasion assay in **(e)** MC38 and **(h)** HCT116 control and knockdown cell lines. Statistical analysis was performed using GraphPad Prism software. Data was analyzed by using one-way ANOVA followed by Newman-Keuls Multiple Comparison Test. P values are represented by stars where: *** P ≤ 0.0001 when compared to the respective non-silencing control treated cell lines.

MMP13 is a part of the breast cancer metastasis signature and tumor cell expression of *MMP13* is linked with increased invasiveness and ability to metastasize in melanoma and breast cancer [18,21]. Immunohistochemical analysis of murine experimental MC38 colon carcinoma tumors in the liver and human colorectal cancer metastases to the liver show MMP13 staining within these tumors (Figure 4a, b). We evaluated *Mmp13* gene expression in murine and human colorectal cancer cell lines and found that *Mmp13* is expressed by the MC38 (murine) and HCT116 (human) colorectal cancer cell lines. To assess the role of tumor derived MMP13 on the ability of tumor cells to metastasize to the liver, we developed MMP13 stable knockdown cell lines (Additional file 1: Figure S4) using RNA interference. *In vitro*, knockdown of *Mmp13* does not have a significant effect on cell proliferation as determined by the MTT assay in the MC38 nor the HCT116 cell line (Figure 4c, f). To study the effect of MMP13 on cell migration, control and *Mmp13* knockdown cells were seeded in a modified Boyden chamber. Both MC38 and HCT116 cell lines showed a decrease in transwell migration compared with the respective control cells (Figure 4d, g). Next, the knockdown cell lines were evaluated for their ability to invade through matrigel with the modified Boyden chamber and demonstrated a decreased invasive ability with the knockdown of *Mmp13* in both cell lines (Figure 4e, h). Data was analyzed by using one-way ANOVA followed by Newman-Keuls multiple comparison test. P values are represented by stars where: *** P ≤ 0.0001 when compared to the respective non-silencing control treated cell lines. For the HCT116 cell line, there was an average 9 fold decrease in transwell invasive ability between the knockdown cell lines and controls, and a 2.5 fold decrease in transwell migration, suggesting loss of MMP13 effects cell invasion in addition to its effect on cell migration. These results suggest that tumor derived MMP13 is essential for migration as well as invasion.

### Loss of tumor derived MMP13 reduces the ability of tumor cells to metastasize in vivo

**Figure 5:**
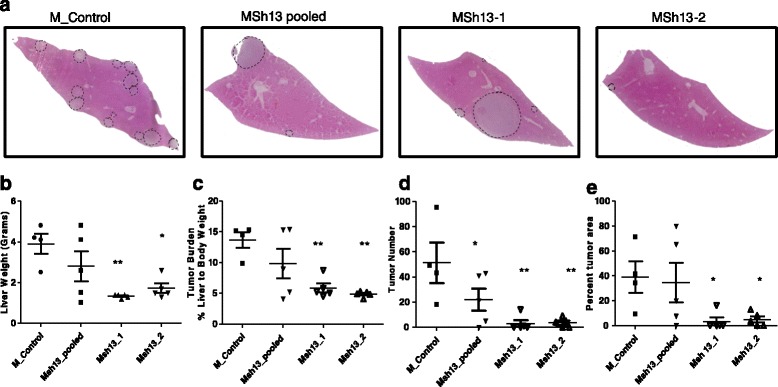
**Knockdown of tumor derived MMP13 leads to decreased tumor metastasis to the liver. (a)** Representative liver cross sections stained with Haematoxylin & Eosin of wildtype mice injected with control or MMP13 knockdown MC38 cell lines (MSh13 pooled, MSh13-1, and MSh13-2). Dashed black lines denote tumors. Quantification of metastatic tumor burden measured by **(b)** liver weight, **(c)** tumor burden as a percent liver weight to total body weight, **(d)** metastatic seeding by quantification of tumor number and **(e)** percentage of tumor area to total liver area in normal and steatotic livers (n = 5 per group). Statistical analysis was performed using GraphPad Prism software. Data was analyzed by using one-way ANOVA followed by Newman-Keuls Multiple Comparison Test. P values are represented by stars where: * ≤ .05, ** ≤ .01, and *** ≤ .001 when compared to mice injected with non-silencing control cell lines.

To test the importance of tumor cell derived MMP13 on establishment of metastasis to the liver, we utilized the splenic injection model to deliver 2.5 × 10^5^*Mmp13* knockdown or control MC38 cell lines into wildtype C57bl/6 mice. 21 days post injection, mice were sacrificed and livers harvested. The liver weight and percent liver weight to total body weight ratio was measured to determine metastatic burden (Figure 5b, c). Liver sections were stained by H&E (Figure 5a) to record the incidence (Figure 5c) and area (Figure 5d) of metastasis. Overall, the tumor burden and incidence of metastases decreased in the mice injected with *Mmp13* knockdown cells compared to control cells. Statistical analysis was carried out by one-way ANOVA followed by Newman-Keuls multiple comparison test. P values are represented by stars where: * ≤ .05, ** ≤ .01, and *** ≤ .001 (Figure 5). Liver metastases were stained for Ki67 (proliferation marker) and cleaved caspase-3 (apoptosis marker) to examine the role of MMP13 in promoting the survival and outgrowth of metastatic tumors (Additional file 1: Figure S5). The percentage of tumor cells proliferating or undergoing apoptosis were similar between the wildtype and *Mmp13*−/− tumors of comparable size suggesting no difference in the rate of tumor growth between the WT and *Mmp13*−/− mice. Additionally, there was no difference in tumor vascularity as determined by vWF staining (Additional file 1: Figure S5). These results suggest that tumor derived MMP13 facilitates the establishment of metastases in the liver without affecting metastatic outgrowth.

## Discussion

The liver is a common site of metastasis for several types of cancer [26] and identification of molecular effectors that can prevent metastasis to the liver is of important clinical relevance. NAFLD is increasingly becoming recognized as the most common cause of liver disease and it is associated with increased risk of development of primary liver cancers, even prior to establishment of cirrhosis [1]. Progression of NAFLD results in changes in the liver microenvironment and alterations in the extracellular matrix, which effects cancer progression and outcome [27,28]. The MMPs are an important class of proteases that can alter the extracellular matrix and influence the microenvironmental integrity. There is considerable evidence supporting the role they play at different steps of malignant tumor metastasis including tumor cell intravasation and extravasation [29]. We initially evaluated the steatotic microenvironment for alterations in the gene expression levels for a panel of MMPs that have been previously associated with cancer progression. Our results demonstrate that MMP13, an interstitial collagenase, is elevated in the steatotic liver in our murine model as well as in human patient samples with NAFLD. Other groups have additionally shown that MMP13 is elevated in fibrotic liver disease [16] and could be relevant in other settings such as hepatitis as well. Several studies also link elevated levels of MMP13 in either the stroma or tumor cells with cancer progression [17,18,20,21], however there are reports that alternately suggest a protective role for MMP13 [30,31]. There still remains a limited understanding of the role of MMP13 in the liver microenvironment and its influence on the establishment of hepatic metastases. Here, we evaluated the role of both stromal and tumor cell derived MMP13 on the establishment of metastases in the liver.

Using mice genetically deficient in MMP13 we have shown that loss of stromal MMP13 leads to a significant decrease in the tumor burden in the liver. This effect was seen in both normal and steatotic livers, suggesting that elevation of MMP13 in the liver plays a role in tumor metastasis. Although we saw a significant decrease in tumor burden in the *Mmp13* deficient mice, differences in proliferation or apoptosis were not observed within the tumors, nor were any differences in the vascularity of the tumors. This suggested that the difference in metastatic burden was due to early events in the metastatic cascade. Similar levels of metastatic cell dissemination and early cell survival were observed in wildtype compared to *Mmp13* deficient livers, yet the loss of host MMP13 diminished the ability of tumor cells to extravasate. Several factors can influence the ability of tumor cells to adhere to the vascular walls and extravasate into the surrounding tissues. Studies have shown that changes in expression level and structure of collagen surrounding the vasculature are important in the ability of tumor cells to extravasate [31-33]. Since MMP13 is a collagenase, we evaluated the level of collagen I, II and IV in the liver but found that mice lacking MMP13 had no significant differences in collagen mRNA expression and immunofluorescence staining pattern compared to the wildtype mice. Further, we did not observe any differences in fibrotic disease progression as shown by trichrome staining of murine liver sections (Additional file 1: Figure S6).

Although we did not observe changes in collagen in the liver, MMP13 may mediate release or activation of critical factors involved in tumor cell extravasation. MMP13 can cleave, release and activate cytokines thereby altering recruitment and or activation of inflammatory cells such as neutrophils and macrophages that have been shown to facilitate tumor cell extravasation [34-37]. We evaluated changes in the lymphoid and myeloid lineage inflammatory cell sub populations within the liver of wildtype and *Mmp13*−/− mice but did not see significant differences within the inflammatory cell subpopulations. However, the activation state and function of the immune cells could alternately explain the differences in tumor burden observed without being reflected in absolute cell numbers.

MMP13 expression has been observed in invasive malignant tumors such as breast carcinomas, squamous cell carcinomas (SCCs) of the head and neck and vulva, primary and metastatic melanomas, hepatocellular carcinoma cell lines [38] and transitional cell carcinoma of the urinary bladder [39]. Colorectal cancers can be classified as either the most commonly seen classical nonmucinous adenocarcinomas (AC) or as the less frequent subtypes of mucinous adenocarcinomas (MAC) and signet-ring cell carcinomas (SC) [40]. Recent studies showed that the nonmucinous ACs had a higher tumor derived MMP13 protein levels than MACs and SCs, which are more invasive and have a higher frequency of lymph node metastasis than nonmucinous ACs [41]. MACs and SCs have different characteristics from classical ACs and might have additional mutational changes that circumvent the requirement of MMP13 and affect their ability to invade and metastasize [40]. Further, levels of related MMPs including MMP1, MMP8, MMP14, MMP2 and MMP9 which have previously been shown to affect tumor cell invasion were not evaluated in these studies and could compensate for MMP13 levels [42]. Our results indicate that both the MC38 murine colon cancer line and the HCT116 colon cancer cells express MMP13. Knockdown of *Mmp13* utilizing lentiviral shRNA resulted in a decreased ability of tumor cells to migrate and invade *in vitro* and correspondingly led to the development of fewer metastasis *in vivo*. Coupled together, both tumor cell and host derived MMP13 promote the establishment of metastases in the liver.

The use of selective MMP13 inhibitors may be an important step to control tumor growth and metastasis to the liver, however in the past, the clinical use of MMP inhibitors has been hampered by their lack of specificity [43]. Fortunately, progress is being made toward developing specific inhibitors and a few MMP13 selective inhibitors are currently being studied [19,44]. MMP13 is an ideal candidate with relatively low expression in normal adult tissues that will limit unwanted side effects when targeted [15]. It will therefore be important to determine whether MMP13 selective inhibitors can pharmacologically block metastasis to the liver.

## Conclusions

In conclusion, we have shown that MMP13 is elevated in the setting of hepatic steatosis and that both tumor and stromal derived MMP13 are involved in attenuating metastatic tumor burden in the liver. Collectively, these data suggest that MMP13 could represent a new therapeutic target in the management of metastasis to the liver.

## Methods

### Patient samples

Liver biopsies from consented patients undergoing bariatric surgery were collected and flash frozen for protein or RNA collection and further de-identified in accordance with a protocol approved by Vanderbilt’s Internal Review Board (VUMC IRB#120829). Samples were reviewed by a Vanderbilt University pathologist and classified as either Normal (<5% Steatosis), Steatosis, Steatohepatitis or NAFLD related cirrhosis based on histology. De-identified formalin-fixed, paraffin-embedded liver tissue sections from patients with colorectal cancer metastasis to the liver were obtained from the Vanderbilt Translational Pathology Shared Resource.

### Mice

8 week old C57bl/6 J male mice were obtained from Jackson Research Laboratories (Bar Harbor, ME) and a breeding pair of MMP13 null mice was obtained (Laboratory of Dr. Zena Werb, UCSF). Mice were housed in a level 6 animal facility at Vanderbilt University. Mice were fed either regular chow, a 13.5% fat diet (5001, LabDiet) or fed a 42% calories from fat diet (TD.88137, Harlan Teklad) *ad libitum* for 3 months at which time point we have shown that wildtype mice develop prominent steatosis [7].

### Cell lines

MC38 murine colon cancer cells lines, syngeneic to C57Bl/6 background were provided by Dr. Steven Libutti, NCI. HCT116 human colorectal carcinoma cell line was obtained from ATCC (CCL-247). Cell lines were grown in culture conditions of 10% fetal bovine serum (Atlanta Biologicals, Lawrenceville, GA) in Dulbecco’s Modified Eagle Media (Gibco BRL, Carlsbad, CA) at 37°C and 5% CO_2_, and harvested at 75% confluence for experimental studies.

### Western blot

Protein lysates were prepared with RIPA lysis buffer. Protein concentration was determined by the Pierce BCA protein assay (Thermo Scientific). 30 μg of protein was loaded into each well and separated on a 10% SDS-PAGE gel. Proteins were transferred onto a nitrocellulose membrane, subsequently blocked with 3% milk, and then incubated with anti MMP13 antibody (Abcam, ab39012) overnight at 4°C. The blots were washed and then incubated with secondary antibody (IR700 conjugated donkey anti rabbit IgG) for 1 h at room temperature. Blots were washed and then imaged with Licor Odyssey scanner.

### qRT-PCR

RNA was extracted using a combined Qiazol extraction and subsequent Qiagen RNeasy mini kit. One microgram of total RNA was reverse transcribed using the high capacity cDNA reverse transcription kit (Applied Biosystems) and real-time PCR was performed using specific primers for mouse MMPs with GAPDH as control (Qiagen, QT00116116, QT00107751, QT00110012, QT00113540, QT00108815, QT00115521, QT00099729, QT01658692, QT00098945, QT01064308). Real-time PCR was performed with the iQ SYBR green supermix kit (Bio-Rad) according to the manufacturer’s instructions and measured via a CFX96 real time PCR detection system (Bio-Rad). Experiments were done in triplicate with three replicates per sample. Fold-change was determined relative to normal wildtype samples and calculated using GAPDH levels as a reference.

### Histology and immunohistochemistry

Liver tissue was formalin fixed, embedded in paraffin blocks, and cut into 6 μm sections. For histology, sections were rehydrated with xylenes and a decreasing ethanol series and then stained with Mayer’s Hematoxylin (Sigma) and Eosin. Hydrated sections were boiled in a citric acid solution (10 mM trisodium salt dihydrate pH 6.0, 0.5% Tween-20) for 8 minutes to unmask antigen. Slides were stained with primary antibody (MMP13: sc-12363, Ki67: ab15580, Cleaved caspase 3: cell signaling D175, vWF: Dako A0082) and incubated overnight at 4°C, washed and incubated with biotinylated secondary antibody (Vector Labs), processed with the ABC Vectastain kit (Vector Labs) and developed in chromogen solution (0.1 M Tris–HCl pH 7.4, 1.125 mM diaminobenzidine, 0.01% H_2_O_2_). Slides were counterstained with Mayer’s Hematoxylin Solution (Sigma), dehydrated with ethanols and mounted with permount. Slides were imaged with a Q Imaging Micropublisher color digital camera mounted to a Zeiss Axioplan 2 microscope using MetaMorph software for acquisition.

### Experimental liver metastasis

Experimental liver metastasis was carried out as previously described [7]. Briefly, 5-month-old wildtype or *Mmp13* knockout mice that had been on diet for 3 months were injected with 5 × 10^5^ MC38 parental or *Mmp13* knockdown cells into the spleen and allowed to perfuse to the liver for 3 minutes before splenectomy. Mice were maintained on respective diets and sacrificed 14 days post-injection. At the time of sacrifice the mice were weighed, the livers were removed and weighed, and the livers were processed for histology as described above. Graphical representation of metastatic burden was calculated using GraphPad software to compare total liver weights and percent liver weight relative to total animal weight. Further, in-depth quantitative analysis of tumor burden was assessed on the left lateral lobe of the liver, that was fixed and cut sagittally into four parts, paraffin-embedded, sectioned and stained for H&E. Slides were scanned at 20X using an Ariol® SL-50 scanner. Ariol software was used to quantify the size and number of tumors per section.

### Tumor cell extravasation

To determine the number of tumor cells extravasating in the liver, we utilized the methodology previously described by Martin et al. [25], with some modifications. Briefly, mice were injected intrasplenically with 1×10^6^ cell tracker red (Life Technologies) labeled MC38 tumor cells. At 24 and 48 hours post injection, mice were anesthetized using isoflurane and placed on mechanical ventilation at 60 breaths/minute through surgical tracheostomy. 100 μl of 0.5 mg/ml 488-tomato lectin vascular label (Vector Laboratories, DL-1174) was injected into the spleen and allowed to circulate throughout the body for 6 minutes. Next, the abdominal aorta was cut to provide outflow and livers were perfused with PBS using gravity pressure through the heart and spleen. After the effluent had cleared, the liver was resected en-bloc, placed in a glass bottom dish with #1.5 glass (In Vitro Scientific) and immediately imaged.

Images were acquired with a LSM780 confocal microscope (Carl Zeiss Inc.) using a Fluar 40X oil objective with NA = 1.30 at room temperature. 488 nm and 561 nm laser lines were used to simultaneously excite fluorescence from the vasculature cell tracker labeled tumor cells. The band-pass emission filters for the two channels were set as follows: 499 – 552 nm for the green fluorescence, and 602 – 747 nm for the red fluorescence. The pixel size was set to 0.692 μm and the dwell time was 12.6 μs. Images were acquired every 0.700 μm for every z-stack. The laser power of the laser lines was independently adjusted for each stack, between 0.1% and 3%, to avoid saturated pixels and to maintain a good signal to noise ratio through all the planes. The bit depth of the images was set to 12 bit. Image analysis was performed using Fiji [45]. The tridimensional structure of the vasculature was reconstructed from each z-stack using the Tubeness function [46] and overlaid onto the red channel to identify a cell as extravasating or not. Percentage of cells extravasating at each time point was determined from over 30 individual tumor cells per mouse (n = 3 per group).

### Establishment of stable MMP13 knockdown cell lines

Pooled lentiviral particles targeting three different regions of murine *Mmp13* (Open Biosystems: V2LMM_28573, V2LMM_ 34601, V2LMM_37490) and control particles were used to transfect MC38 cells. Control non-target and specific human *MMP13* shRNA lentiviral particles targeting three different regions of human *MMP13* (sc-41559) were used to transfect HCT116 cells. Post-transfection, shRNA-expressing cells were selected with puromycin. Knockdown was confirmed in resulting clones with qRT-PCR and western blot analysis.

### MTT

Proliferation of cells was determined using the MTT assay. Briefly, 10×10^4^ cells were plated in each well of a 96 well plate and allowed to attach overnight. Cells were serum starved for 24 hours followed by changing to DMEM containing 10% FBS for 24 hours. 20 μl of MTT reagent (5 mg/ml, Sigma) was added to each well and incubated at 37°C for 2 hours after which the media was aspirated and the remaining MTT formazan crystals dissolved in 100 μl of isopropanol. Absorbance at 570 nm was read using a Victor3 V 1420 Multilabel Plate Counter. Experiments were carried out in triplicate with 5 replicates per plate.

### Transwell migration and invasion assays

Cell migration and invasion were assessed by a modified Boyden assay using 24 well multi-well inserts (BD) with 8 μm pore PET membrane. *Mmp13* knockdown and control MC38 or HCT116 cells (1×10^5^ cells/chamber) were seeded in DMEM plus 1% FBS directly on the insert membrane (Migration) or resuspended in 100 μl of 1 mg/ml of basement membrane matrix (BD) (Invasion) and seeded on the membrane. DMEM with 10% FBS was added to the lower chamber and cells were allowed to migrate through the filter for 16 h at 37°C in 5% CO_2_ for MC38 cells and 48 h for HCT116 cell lines. MC38 cells were allowed to invade for 24 h and HCT116 cells for 72 hours. Cells on the lower surface of the membrane were fixed in 100% methanol, stained with Dapi, and imaged. Experiments were carried out in triplicate with three replicates per experiment and the number of cells migrated/invaded per 10X field was determined from five random fields per well.

All animal experimental procedures and protocols were carried out in accordance to the ARRIVE guidelines. Experimental procedures and protocols were approved by the Vanderbilt university medical center IACUC protocol #M/09/216 and performed according to the institutional ethical guidelines for animal care and use. Human tissue samples were collected under the National Cancer Institute (NCI) best practices and CHTN standard operating procedures and approved by the Vanderbilt Internal Review Board (VUMC IRB #120829).

## Additional file

Additional file 1: Figure S1. Characterization of MMP13 -/- mice. Wildtype and MMP13 deficient mice were fed regular or high fat diet for 3 months to determine overall body weight and liver weight. **Figure S2.** Affect of loss of host MMP13 on tumor proliferation, apoptosis and vascularity. **Figure S3.** Tumor size distribution in Wildtype and MMP13-/- mice. **Figure S4.** Establishment of stable MMP13 knockdown cell lines. **Figure S5.** Affect of loss of tumor derived MMP13 on tumor proliferation, apoptosis and vascularity. **Figure S6.** Representative images of trichrome staining of Wildtype and MMP13-/- mice with and without steatosis.

## References

[1] Argo CK, Caldwell SH (2009). Epidemiology and natural history of non-alcoholic steatohepatitis. Clin Liver Dis.

[2] Peverill W, Powell LW, Skoien R (2014). Evolving concepts in the pathogenesis of NASH: beyond steatosis and inflammation. Int J Mol Sci.

[3] Vansaun MN, Mendonsa AM, Lee Gorden D (2013). Hepatocellular proliferation correlates with inflammatory cell and cytokine changes in a murine model of nonalchoholic fatty liver disease. PLoS One.

[4] Bugianesi E (2007). Non-alcoholic steatohepatitis and cancer. Clin Liver Dis.

[5] Schütte K, Bornschein J, Malfertheiner P (2009). Hepatocellular carcinoma–epidemiological trends and risk factors. Dig Dis.

[6] Polednak AP (2008). Estimating the number of U.S. incident cancers attributable to obesity and the impact on temporal trends in incidence rates for obesity-related cancers. Cancer Detect Prev.

[7] VanSaun MN, Lee IK, Washington MK, Matrisian L, Gorden DL (2009). High fat diet induced hepatic steatosis establishes a permissive microenvironment for colorectal metastases and promotes primary dysplasia in a murine model. Am J Pathol.

[8] Spano D, Heck C, De Antonellis P, Christofori G, Zollo M (2012). Molecular networks that regulate cancer metastasis. Semin Cancer Biol.

[9] Yu Q, Stamenkovic I (2000). Cell surface-localized matrix metalloproteinase-9 proteolytically activates TGF-beta and promotes tumor invasion and angiogenesis. Genes Dev.

[10] Rundhaug JE (2005). Matrix metalloproteinases and angiogenesis. J Cell Mol Med.

[11] Egeblad M, Werb Z (2002). New functions for the matrix metalloproteinases in cancer progression. Nat Rev Cancer.

[12] Sternlicht MD, Werb Z (2001). How matrix metalloproteinases regulate cell behavior. Annu Rev Cell Dev Biol.

[13] Decock J, Thirkettle S, Wagstaff L, Edwards DR (2011). Matrix metalloproteinases: protective roles in cancer. J Cell Mol Med.

[14] Coussens LM, Fingleton B, Matrisian LM (2002). Matrix metalloproteinase inhibitors and cancer: trials and tribulations. Science.

[15] Tardif G, Reboul P, Pelletier J-P, Martel-Pelletier J (2004). Ten years in the life of an enzyme: the story of the human MMP-13 (collagenase-3). Mod Rheumatol.

[16] Uchinami H, Seki E, Brenner DA, D’Armiento J (2006). Loss of MMP 13 attenuates murine hepatic injury and fibrosis during cholestasis. Hepatology.

[17] Yamada T, Oshima T, Yoshihara K, Tamura S, Kanazawa A, Inagaki D (2010). Overexpression of MMP-13 gene in colorectal cancer with liver metastasis. Anticancer Res.

[18] Ellsworth RE, Seebach J, Field LA, Heckman C, Kane J, Hooke JA (2009). A gene expression signature that defines breast cancer metastases. Clin Exp Metastasis.

[19] Shah M, Huang D, Blick T, Connor A, Reiter LA, Hardink JR (2012). An MMP13-selective inhibitor delays primary tumor growth and the onset of tumor-associated osteolytic lesions in experimental models of breast cancer. PLoS One.

[20] Kominsky SL, Doucet M, Thorpe M, Weber KL (2008). MMP-13 is over-expressed in renal cell carcinoma bone metastasis and is induced by TGF-beta1. Clin Exp Metastasis.

[21] Zigrino P, Kuhn I, Bäuerle T, Zamek J, Fox JW, Neumann S (2009). Stromal expression of MMP-13 is required for melanoma invasion and metastasis. J Invest Dermatol.

[22] Gorden DL, Fingleton B, Crawford HC, Jansen DE, Lepage M, Matrisian LM (2007). Resident stromal cell-derived MMP-9 promotes the growth of colorectal metastases in the liver microenvironment. Int J Cancer.

[23] Deryugina EI, Quigley JP (2006). Matrix metalloproteinases and tumor metastasis. Cancer Metastasis Rev.

[24] De Meijer VE, Sverdlov DY, Le HD, Popov Y, Puder M (2012). Tissue-specific differences in inflammatory infiltrate and matrix metalloproteinase expression in adipose tissue and liver of mice with diet-induced obesity. Hepatol Res.

[25] Martin MD, Kremers G-J, Short KW, Rocheleau JV, Xu L, Piston DW (2010). Rapid extravasation and establishment of breast cancer micrometastases in the liver microenvironment. Mol Cancer Res.

[26] Leong SPL, Cady B, Jablons DM, Garcia-Aguilar J, Reintgen D, Jakub J (2006). Clinical patterns of metastasis. Cancer Metastasis Rev.

[27] Cohen JC, Horton JD, Hobbs HH (2011). Human fatty liver disease: old questions and new insights. Science.

[28] Starley BQ, Calcagno CJ, Harrison SA (2010). Nonalcoholic fatty liver disease and hepatocellular carcinoma: a weighty connection. Hepatology.

[29] Kessenbrock K, Plaks V, Werb Z (2010). Matrix metalloproteinases: regulators of the tumor microenvironment. Cell.

[30] Fukuda H, Mochizuki S, Abe H, Okano HJ, Hara-Miyauchi C, Okano H (2011). Host-derived MMP-13 exhibits a protective role in lung metastasis of melanoma cells by local endostatin production. Br J Cancer.

[31] Perry SW, Schueckler JM, Burke K, Arcuri GL, Brown EB (2013). Stromal matrix metalloprotease-13 knockout alters Collagen I structure at the tumor-host interface and increases lung metastasis of C57BL/6 syngeneic E0771 mammary tumor cells. BMC Cancer.

[32] Shintani Y, Hollingsworth MA, Wheelock MJ, Johnson KR (2006). Collagen I promotes metastasis in pancreatic cancer by activating c-Jun NH(2)-terminal kinase 1 and up-regulating N-cadherin expression. Cancer Res.

[33] Lu J, Zhou S, Siech M, Habisch H, Seufferlein T, Bachem MG (2014). Pancreatic stellate cells promote hapto-migration of cancer cells through collagen I-mediated signalling pathway. Br J Cancer.

[34] Morrison C, Mancini S, Cipollone J, Kappelhoff R, Roskelley C, Overall C (2011). Microarray and proteomic analysis of breast cancer cell and osteoblast co-cultures: role of osteoblast matrix metalloproteinase (MMP)-13 in bone metastasis. J Biol Chem.

[35] Dufour A, Overall CM (2013). Missing the target: matrix metalloproteinase antitargets in inflammation and cancer. Trends Pharmacol Sci.

[36] Spicer JD, McDonald B, Cools-Lartigue JJ, Chow SC, Giannias B, Kubes P (2012). Neutrophils promote liver metastasis via Mac-1-mediated interactions with circulating tumor cells. Cancer Res.

[37] Auguste P, Fallavollita L, Wang N, Burnier J, Bikfalvi A, Brodt P (2007). The host inflammatory response promotes liver metastasis by increasing tumor cell arrest and extravasation. Am J Pathol.

[38] Yang Z, Zhang Y, Wang L (2013). A feedback inhibition between miRNA-127 and TGFβ/c-Jun cascade in HCC cell migration via MMP13. PLoS One.

[39] Reunanen N, Kähäri V: Matrix metalloproteinases in cancer cell invasion. In: Madame Curie bioscience database; Austin (TX): Landes Bioscience; 2000

[40] Nitsche U, Zimmermann A, Späth C, Müller T, Maak M, Schuster T (2013). Mucinous and signet-ring cell colorectal cancers differ from classical adenocarcinomas in tumor biology and prognosis. Ann Surg.

[41] Foda AA-RM, El-Hawary AK, Abdel-Aziz A (2013). Differential expression of matrix metalloproteinase-13 in mucinous and nonmucinous colorectal carcinomas. Ann Diagn Pathol.

[42] Stamenkovic I (2000). Matrix metalloproteinases in tumor invasion and metastasis. Semin Cancer Biol.

[43] Dormán G, Cseh S, Hajdú I, Barna L, Kónya D, Kupai K (2010). Matrix metalloproteinase inhibitors: a critical appraisal of design principles and proposed therapeutic utility. Drugs.

[44] Li N-G, Shi Z-H, Tang Y-P, Wang Z-J, Song S-L, Qian L-H (2011). New hope for the treatment of osteoarthritis through selective inhibition of MMP-13. Curr Med Chem.

[45] Schindelin J, Arganda-Carreras I, Frise E, Kaynig V, Longair M, Pietzsch T (2012). Fiji: an open-source platform for biological-image analysis. Nat Methods.

[46] Sato Y, Nakajima S, Shiraga N, Atsumi H, Yoshida S, Koller T (1998). Three-dimensional multi-scale line filter for segmentation and visualization of curvilinear structures in medical images. Med Image Anal.

