# Supplementary material for: Host and tumor derived MMP13 regulate extravasation and establishment of colorectal metastases in the liver
Source: Mol Cancer. 2015 Feb 22;14:49. doi: 10.1186/s12943-014-0282-0 (PMC4351934; doi:10.1186/s12943-014-0282-0)
Supplement: Additional file 1: Figure S1. — Characterization of MMP13 -/- mice. Wildtype and MMP13 deficient mice were fed regular or high fat diet for 3 months to determine overall body weight and liver weight. Figure S2. Affect of loss of host MMP13 on tumor proliferation, apoptosis and vascularity. Figure S3. Tumor size distribution in Wildtype and MMP13-/- mice. Figure S4. Establishment of stable MMP13 knockdown cell lines. Figure S5. Affect of loss of tumor derived MMP13 on tumor proliferation, apoptosis and vascularity. Figure S6. Representative images of trichrome staining of Wildtype and MMP13-/- mice with and without steatosis. [file 12943_2014_282_MOESM1_ESM.pdf]

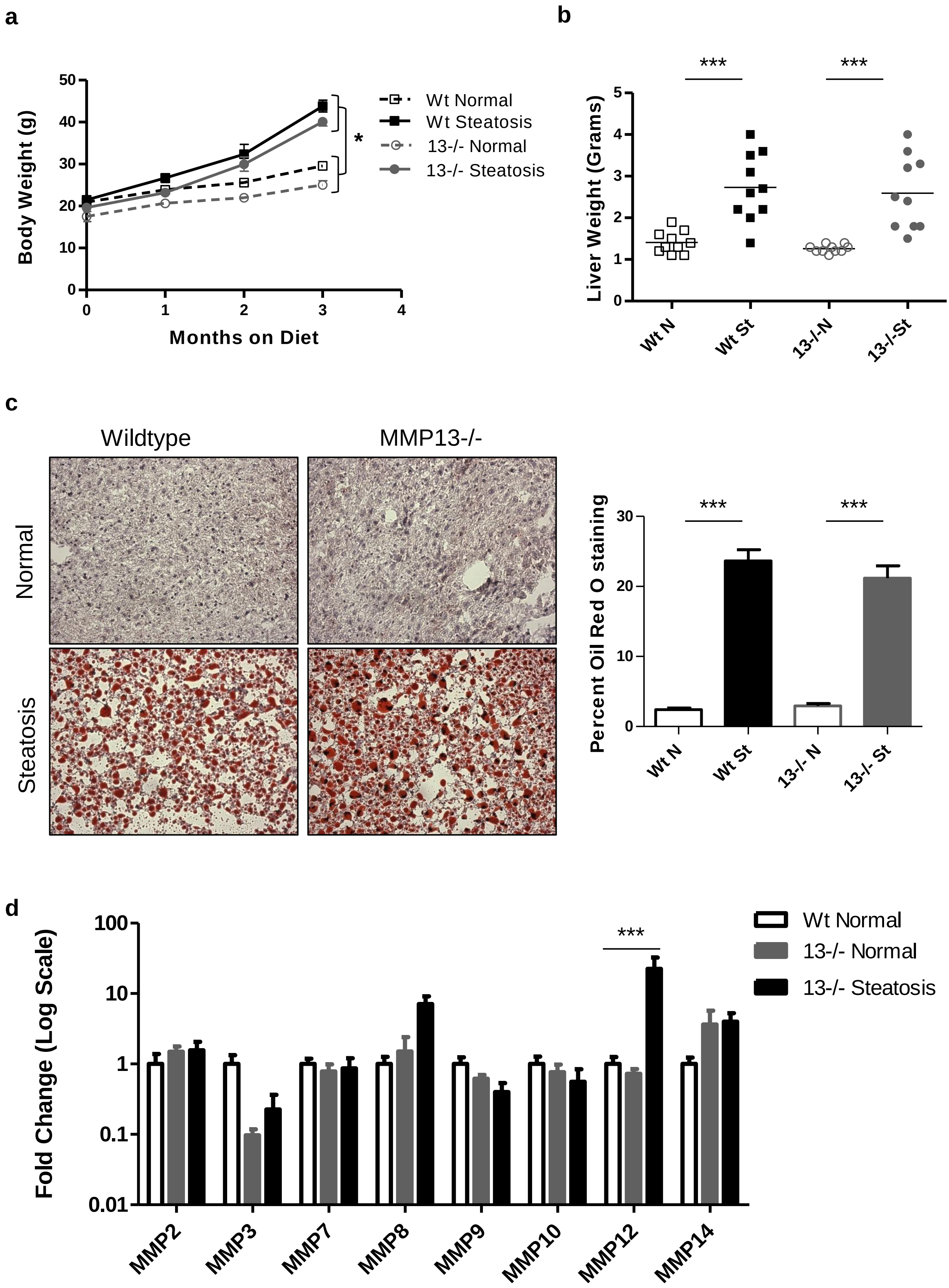

Supplementary Figure S1. Characterization of MMP13 -/- mice

Supplementary Figure S1. Characterization of MMP13 -/- mice. Wildtype and MMP13 deficient mice were fed regular or high fat diet for 3 months to determine overall body weight and liver weight. (a) Graph illustrating total body weight over time with respect to diet and genetic status show that MMP13-/- mice gain weight similar to wildtype mice (n=5). Statistical analysis was carried out using two-way ANOVA followed by Bonferroni post tests to compare different mice groups at each time point. No significant differences were observed between wildtype and MMP13-/- mice on regular chow or on HFD, however both wildtype and MMP13-/- mice have a significant increase in body weight when on HFD after 2 and 3 months indicating that MMP13-/- mice gain weight similar to wildtype animals (b) Graph illustrating total liver weight after 3 months on diet, which trends the same as body weight (n=10). Statiscal analysis was carried out using one way ANOVA followed by Newman-Keuls Multiple Comparison Test (c) Lipid accumulation as visualized as red staining via Oil Red O lipid dye and graphic quantification (n=10). Statiscal analysis was carried out using one way ANOVA followed by Newman-Keuls Multiple Comparison Test (d) Relative gene expression of matrix metalloproteinases (MMPs) in liver tissue of MMP13-/- mice with or without steatosis compared to normal wildtype mice. Relative transcript levels were normalized to GAPDH and expressed as fold change relative to normal controls. Values represent the mean (n = 3). Statistical analysis was carried out using two-way ANOVA followed by Bonferroni post tests to compare changes to Wildtype normal livers. MMP12 was elevated in the setting of steatosis in MMP13-/- mice similar to wildtype mice.

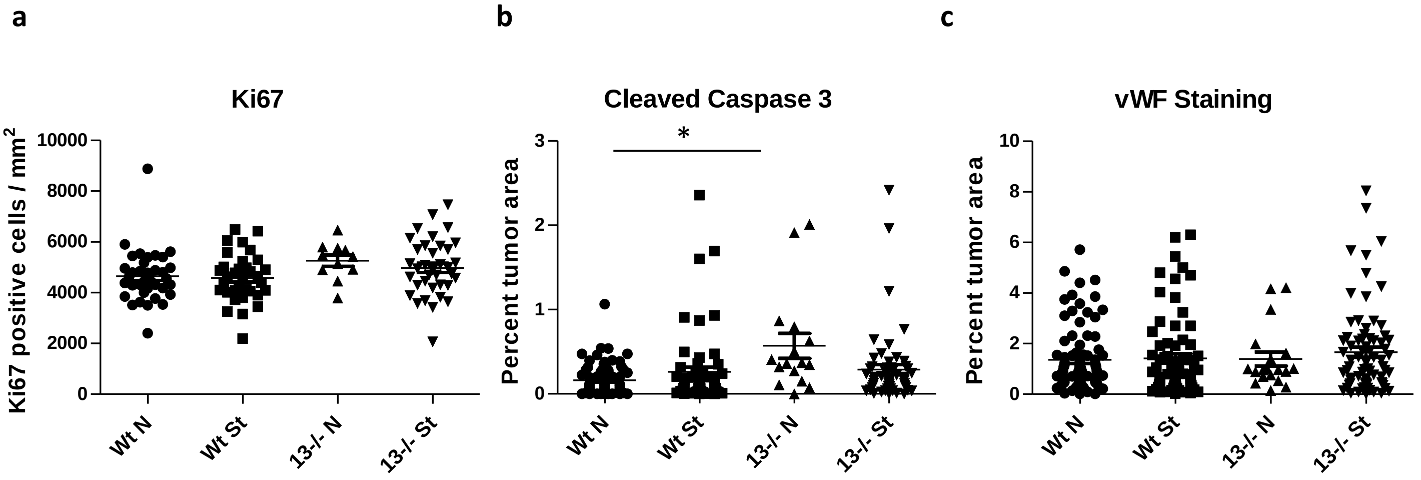

Supplementary Figure S2. Affect of loss of host MMP13 on tumor proliferation, apoptosis and vascularity. (a) Quantification of tumor proliferation by Ki67 staining determined by plotting number of Ki67 positive cells per unit tumor area. (b) Change in apoptosis within tumors in wildtype and MMP13-/- mice determined by percentage of cleaved caspase 3 staining per unit tumor area. (c) Quantification of tumor vascularity as determined by percentage area of von Willebrand factor staining per unit tumor area. Quantification was done using Metamorph software. Statiscal analysis was carried out using one way ANOVA followed by Newman-Keuls Multiple Comparison Test

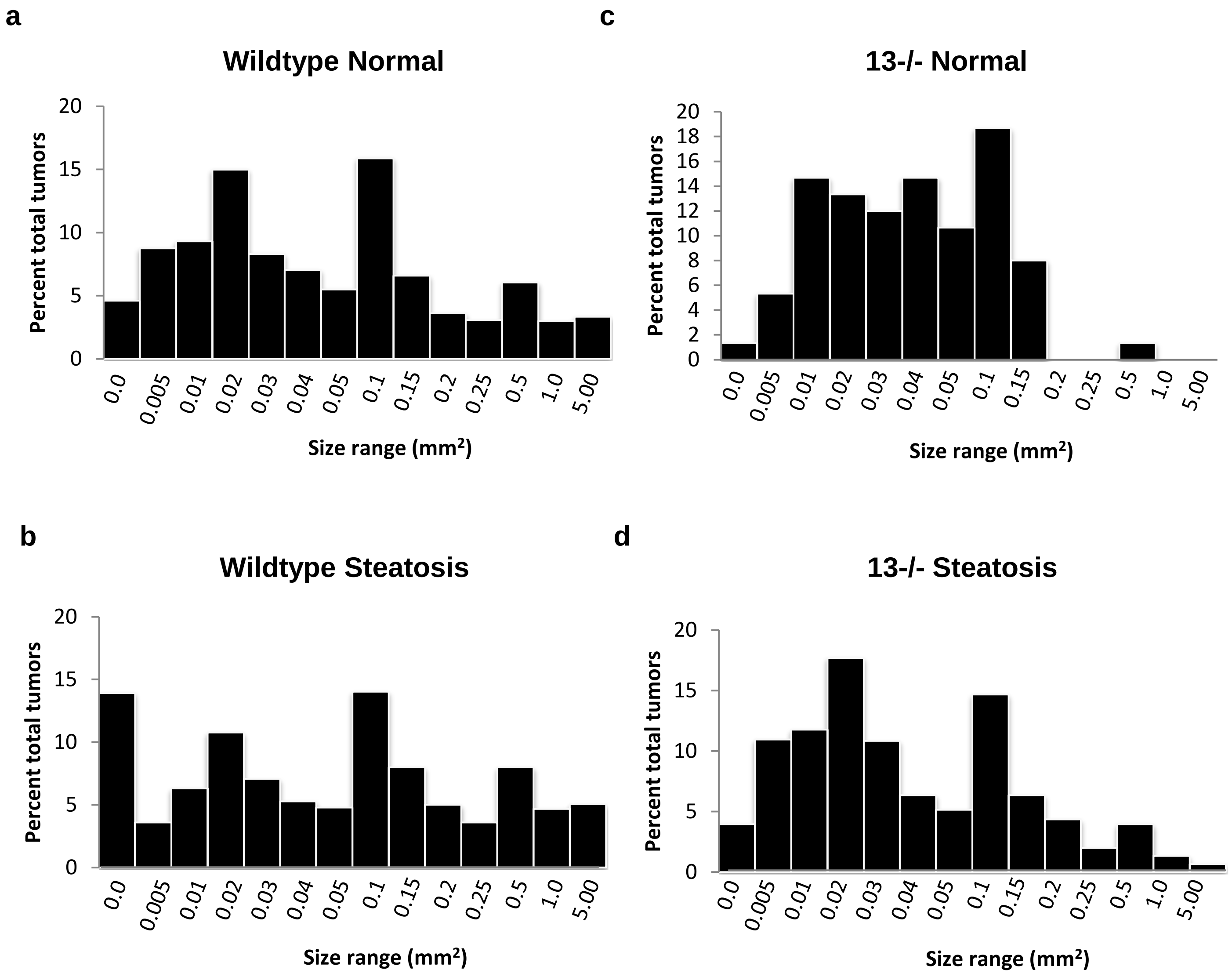

Supplementary Figure S3. Tumor size distribution in Wildtype and MMP13-/- mice. Histogram of MC38 tumor size distribution plotted as a percentage of total number of tumors in (a) wildtype normal, (b) wildtype steatotic, (c) MMP13-/- normal and (d) MMP13-/- steatotic mice.

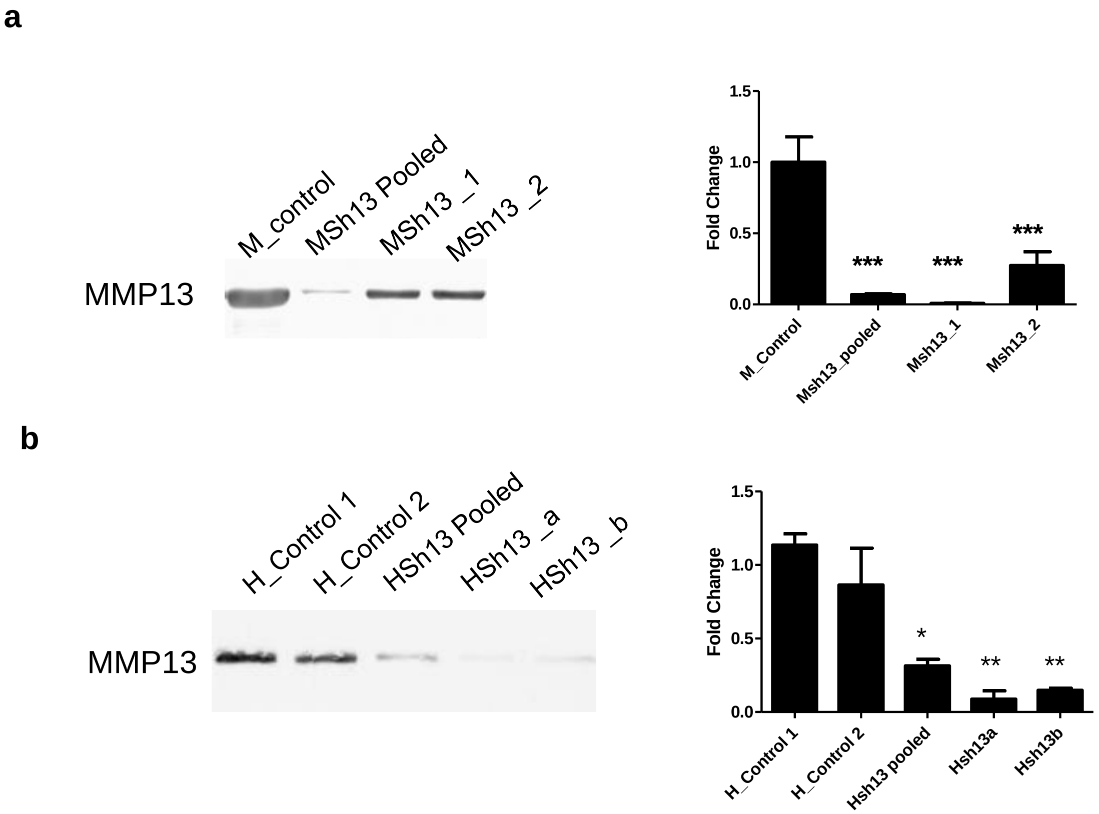

Supplementary Figure S4. Establishment of stable MMP13 knockdown cell lines. (a) Western blot of MMP13 protein levels secreted into the media and quantification of mRNA expression of MMP13 in MC38 control and knockdown cell lines. (b) Western blot of MMP13 protein levels secreted into the media and quantification of mRNA expression of MMP13 in HCT116 control and knockdown cell lines. Statiscal analysis was carried out using one way ANOVA followed by Newman-Keuls Multiple Comparison Test

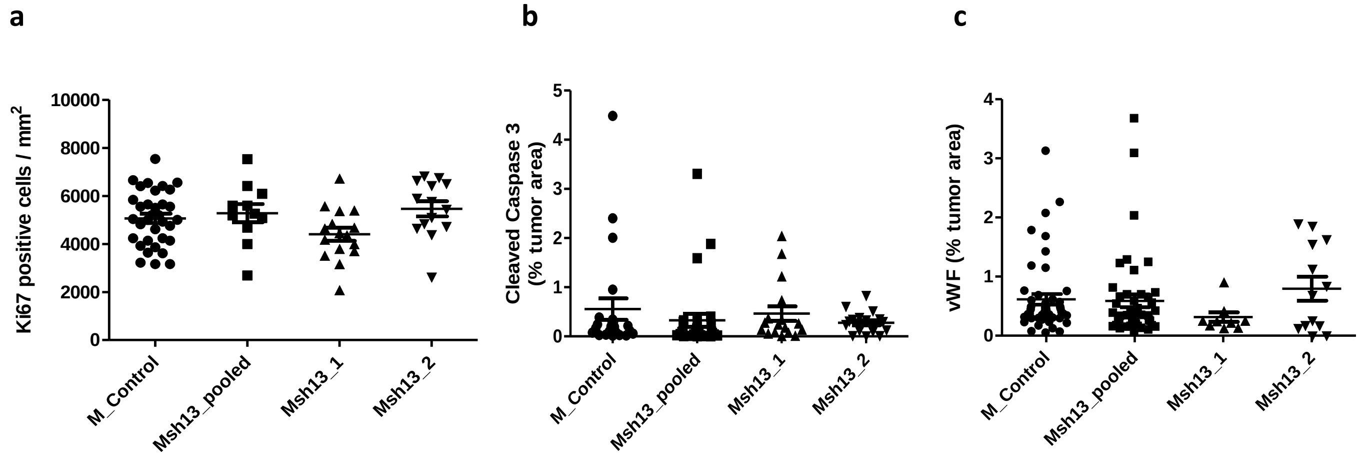

Supplementary Figure S5. Affect of loss of tumor derived MMP13 on tumor proliferation, apoptosis and vascularity. (a) Quantification of tumor proliferation by Ki67 staining determined by plotting number of Ki67 positive cells per unit area. (b) Change in apoptosis within tumors in determined by percentage of Cleaved caspase 3 staining per unit tumor area. (c) Quantification of tumor vascularity as determined by percentage area of von Willebrand factor (vWF) staining per unit tumor area. Quantification was done using Metamorph software. Statiscal analysis was carried out using one way ANOVA followed by Newman-Keuls Multiple Comparison Test

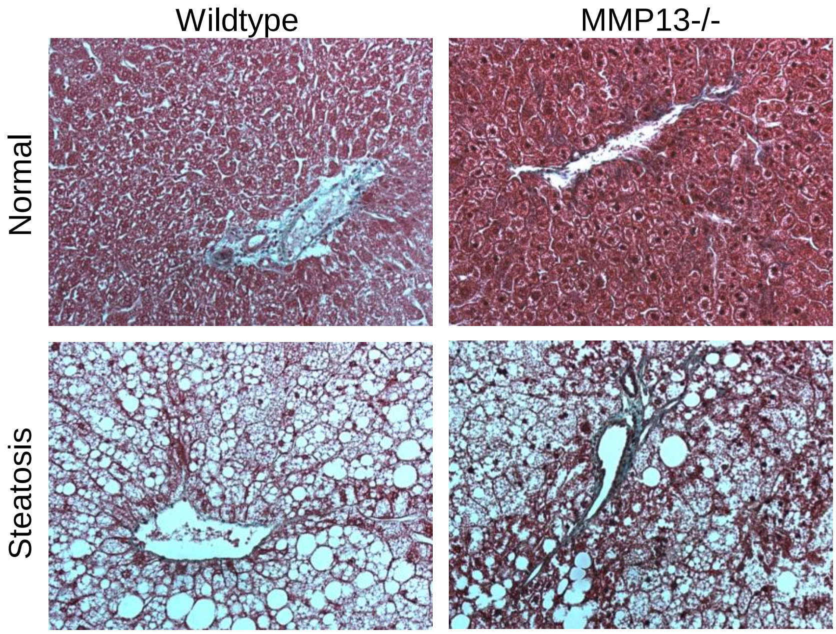

Supplementary Figure S6. Representative images of trichrome staining of Wildtype and MMP13-/- mice with and without steatosis. Collagen accumulation as visualized by blue color via trichrome stain show no stark differences in fibrosis in wiltdtype and MMP13-/- mice.
